# The Neuroprotective Role of Cyanobacteria with Focus on the Anti-Inflammatory and Antioxidant Potential: Current Status and Perspectives

**DOI:** 10.3390/molecules29204799

**Published:** 2024-10-10

**Authors:** Flávia Rodrigues, Mariana Reis, Leonor Ferreira, Clara Grosso, Ricardo Ferraz, Mónica Vieira, Vitor Vasconcelos, Rosário Martins

**Affiliations:** 1 School of Health, Polytechnic Institute of Porto (E2S/P.PORTO), Rua Dr. António Bernardino de Almeida, 400, 4200-072 Porto, Portugal; 10210253@ess.ipp.pt (F.R.); rferraz@ess.ipp.pt (R.F.); mav@ess.ipp.pt (M.V.); 2 Interdisciplinary Centre of Marine and Environmental Research, University of Porto (CIIMAR/CIMAR), Terminal de Cruzeiros do Porto de Leixões, Av. General Norton de Matos s/n, 4450-208 Matosinhos, Portugal; mreis@ciimar.up.pt (M.R.); lferreira@ciimar.up.pt (L.F.); vmvascon@fc.up.pt (V.V.); 3 Department of Biology, Faculty of Sciences, University of Porto (FCUP), Rua do Campo Alegre, Edifício FC4, 4169-007 Porto, Portugal; 4 LAQV/REQUIMTE, School of Engineering, Polytechnic Institute of Porto, Rua Dr. António Bernardino de Almeida, 431, 4249-015 Porto, Portugal; claragrosso@graq.isep.ipp.pt; 5 Center for Translational Health and Medical Biotechnology Research (TBIO/Health Research Network (RISE-Health), E2S/P.PORTO), Rua Dr. António Bernardino de Almeida, 400, 4200-072 Porto, Portugal; 6 LAQV/REQUIMTE, Departamento de Química e Bioquímica, Faculty of Sciences, University of Porto (FCUP), 4169-007 Porto, Portugal

**Keywords:** cyanobacteria, neurodegenerative diseases, antioxidant, anti-inflammatory, neuroprotection, sustainability

## Abstract

Neurodegenerative diseases are linked to the process of neurodegeneration. This can be caused by several mechanisms, including inflammation and accumulation of reactive oxygen species. Despite their high incidence, there is still no effective treatment or cure for these diseases. Cyanobacteria have been seen as a possible source for new compounds with anti-inflammatory and antioxidant potential, such as polysaccharides (sacran), phycobiliproteins (phycocyanin) and lipopeptides (honaucins and malyngamides), which can be interesting to combat neurodegeneration. As a promising case of success, *Arthrospira* (formerly *Spirulina*) has revealed a high potential for preventing neurodegeneration. Additionally, advantageous culture conditions and sustainable production of cyanobacteria, which are allied to the development of genetic, metabolic, and biochemical engineering, are promising. The aim of this review is to compile and highlight research on the anti-inflammatory and antioxidant potential of cyanobacteria with focus on the application as neuroprotective agents. Also, a major goal is to address essential features that brand cyanobacteria as an ecoefficient and economically viable option, linking health to sustainability.

## 1. Introduction

Although difficult to define, neurodegeneration is widely characterized by the progressive dysfunction and death of neurons in the central nervous system (CNS) or in the peripheral nervous system (PNS). The neurodegenerative process is believed to be caused by a set of interconnected mechanisms that include synaptic loss, accumulation of misfolded proteins, mitochondrial dysfunction, chronic inflammation, and excessive amounts of reactive oxygen species (ROS) and reactive nitrogen species (RNS), which cause oxidative stress [1]. Neurodegeneration is associated with a set of neurodegenerative diseases (ND) whose overall symptoms and signals include memory disturbance, dysregulation of movement, namely involuntary movements, and difficulty in speaking and walking [2]. ND is a highly incident, prevalent, and debilitating health condition with a significant impact on individuals, families, and healthcare systems. Highly associated with aging, ND is becoming an increasingly significant problem as the world’s population ages, emphasizing the importance of developing effective therapies and prevention strategies.

The most incident ND include Alzheimer’s disease (AD), Parkinson’s disease (PD), Huntington’s disease (HD), and Amyotrophic lateral sclerosis (ALS) [3]. Briefly, AD is characterized by a slow decline in cognitive abilities, memory loss, and behavioral changes [4]. Although the precise etiology behind AD is unknown, it is thought to be a result of a confluence of hereditary, environmental, and lifestyle factors [4]. Age is considered the primary risk factor for the development of this disease, and it is estimated that around half of the population over the age of 85 can be affected [5]. Moreover, it is anticipated that over 100 million people will have AD by the year 2050 [5]. Brain atrophy in this neurological disease may result from synaptic degradation, neuronal death, amyloid-β (Aβ) plaque deposition, tau protein aggregation and deposition, and inflammatory and oxidative processes [6]. In patients with AD, there is a deficiency in the levels of the neurotransmitters acetylcholine (ACh) and butyrylcholine (BCh) and an overexpression of the enzymes acetylcholinesterase (AChE) and butyrylcholinesterase (BuChE) [7,8]. Therapeutics for AD are typically neuroprotective, anti-inflammatory, and antioxidant [9]. Other treatments include AChE inhibitors such as donepezil, rivastigmine, and galantamine, which have all been approved by the Food and Drugs Administration (FDA) [10]. Memantine is another FDA-approved drug used to treat moderate to severe AD, as it acts as an N-methyl-D-aspartate (NMDA) receptor antagonist. This drug inhibits glutamate hyperactivation, which causes excitotoxicity and neuronal vulnerability [11].

Parkinson’s disease is the second most common ND. Despite age being the main risk factor for this pathology, 5–10% of PD cases are caused by genetic factors that include mutations in the PARKIN and α-synuclein genes [12]. Patients with this disease suffer from bradykinesia (slowness of movement), tremors (typically at rest and absent during movement), rigidity (resistance to movement), and balance changes [12]. This pathology is the result of dopaminergic neuron loss in the substantia nigra pars compacta (SNpc) of the brain. The loss of these neurons is believed to be associated with an increase in ROS production due to inflammation and loss of mitochondrial functions. An unusual aggregation of α-synuclein in Lewy bodies is also observed in PD [13]. The current treatment for PD focuses mainly on the dopaminergic mechanism. The preferred drug for PD treatment is levodopa, which is converted into dopamine in the SNpc. Monoamine oxidase B (MAO-B) inhibitors, which degrade dopamine, are also used in PD therapy [14].

Huntington’s disease is characterized by progressive physical weakening and cognitive decline [15]. HD is an autosomal dominant inherited disease caused by overexpression of the triplet cytosine, adenine, and guanine (CAG) in the huntingtin gene (HTT) on chromosome 4 (4p16.3) [15]. With this mutation, HTT proteins become more susceptible to misfolding, resulting in the formation of aggregates and subsequent deposition, affecting several areas of the brain, especially the striatum [16]. Furthermore, abnormal HTT causes mitochondrial dysfunction, leading to increased production and accumulation of ROS and RNS [17]. Patients suffering from HD show reduced levels of the glucose transporter (GLUT) 1 and 3, increased amounts of lactate, and alterations in the respiratory chain which affect the activity of complexes II, III, and IV [17,18]. Eastern countries such as Japan, Korea, and Hong Kong show a lower prevalence of this condition compared to countries like Canada, United States, and the United Kingdom, which may be related to low mutation rates in these East Asian countries [19]. Despite a correlation between HD and oxidative stress, treatment with antioxidants has not proven effective [17].

Amyotrophic lateral sclerosis, also known as Lou Gehrig’s disease, is a ND that affects upper and lower motor neurons as well as other brain neurons, such as the frontotemporal region [20]. This ND is characterized by weakness, muscle degeneration, and fasciculations. People suffering from this disease normally have a survival time of up to 50 months after diagnosis and usually die from respiratory failure [1,20]. ALS is currently classified as sporadic amyotrophic lateral sclerosis (sALS) or familial amyotrophic lateral sclerosis (fALS). The majority of patients with this disease are included in sALS [21]. In fALS, a large percentage of the cases are caused by mutations in SOD1, C9orf72, TARDBP, and FUS genes [21]. The SOD1 gene, for instance, is important for the modulation of cellular respiration and scavenging excessive superoxide (O_2_^•−^) radicals [22]. ALS leads to various alterations at the cellular level, such as alterations in RNA metabolism, mitochondrial dysfunction, oxidative stress, excitotoxicity, and neuroinflammation [23]. There is no cure for ALS. However, there are drugs that can slow down the progression of this illness and prolong the survival of the patients, such as riluzole, edavarone, sodium phenylbutyrate, and taurursodiol (PB-TURSO) [24,25,26]. New treatments might include the use of stem cells, antibodies, RNA interventions, and small molecules related to oxidative stress and inflammation [26].

Natural compounds have been on the front line as good candidates for neurodegeneration prevention and ND therapies [7]. Within a wide range of organisms, cyanobacteria emerged as a promising source of natural compounds with potential application in specific features of ND, as recently reviewed [7,27]. In fact, several cyanobacterial compounds were found to be active in specific mechanisms of each ND, such as AChE and BChE, inhibition and reduction in Aβ accumulation in AD, and reduction in α-synuclein inclusions in PD.

Although each ND has specific characteristics and affects different areas of the neuronal systems, they share several common features, which include inflammation and oxidative stress, as described above [17]. Cyanobacteria were found to possess strong anti-inflammatory properties that have been shown to impact microglial function, decrease inflammatory mediators, and increase the expression of inflammation-related genes in microglial cells [28]. NDs are also linked to elevated levels of oxidative stress, making the CNS particularly vulnerable due to its high oxidizable substrate content [29]. To protect from oxidative stress, there is substantial evidence that several cyanobacteria genera, namely *Arthrospira,* have strong antioxidant capacity by scavenging ROS, inhibiting lipid peroxidation, and modulating genes related to the oxidative stress response [30].

**Figure 1:**
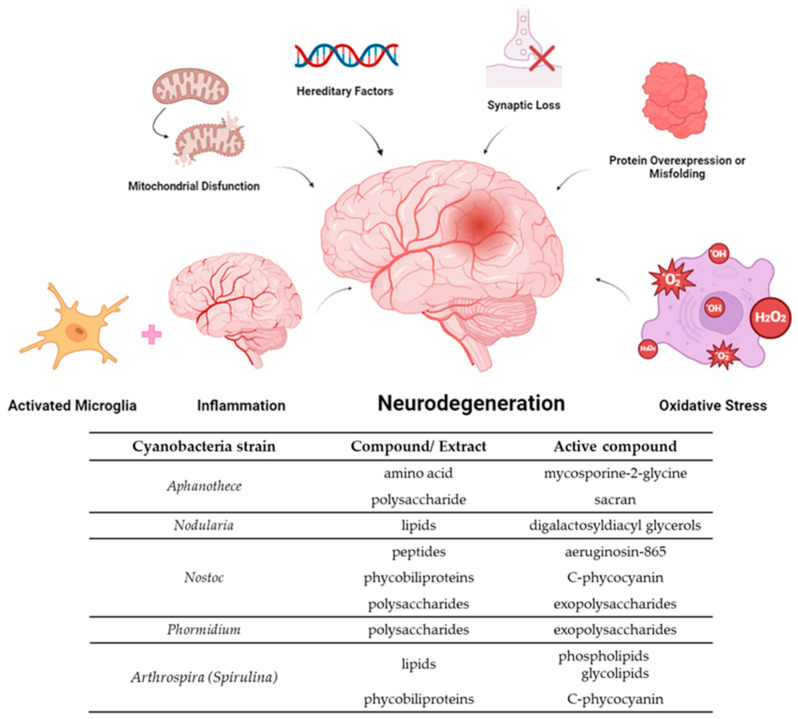
General mechanisms of neurodegeneration and the most promising cyanobacterial natural products for the prevention of neurological disorders include those with well-documented anti-inflammatory and antioxidant properties.

Identifying new compounds to combat inflammation and oxidative stress represents a significant advance in preventing neurodegeneration and, consequently, the onset of the diseases [27,31]. Cyanobacteria have been considered a rich source of extracts and compounds with putative neuroprotective potential [32] (Figure 1). Therefore, the aim of this review is to provide a comprehensive and integrated view of current evidence on the ability of cyanobacteria to prevent and treat neurodegeneration, focusing on the anti-inflammatory and antioxidant potential, to identify existing knowledge gaps and suggest directions for future research. Moreover, considering that the production of natural compounds directed to human health is founded on the culture of the producer organisms, this review highlights the sustainability of cyanobacteria culture, stressing its importance in environmental terms.

## 2. Cyanobacteria

Cyanobacteria, also known as blue-green algae, are Gram-negative prokaryotes and are considered the most primitive photosynthetic organisms on Earth [33]. These are particularly resistant microorganisms that can thrive in a wide range of extreme environments, such as hot springs, frozen systems, and extremely saline environments [33]. These microorganisms can be found in a variety of colors, such as green, red, and yellow [34], due to the presence of natural pigments such as chlorophylls and derivatives, phycobiliproteins (PBPs) and carotenoids that have been used as natural colorants and antioxidants [35].

Cyanobacteria taxonomy has undergone some changes mostly related to the advances in 16S rRNA gene sequencing. In the last years, more than 270 species in 140 genera were newly described with an actual number of 19 orders [36]. Cyanobacteria can have different morphologies; they can be found in unicellular, colonial, and multicellular filamentous forms [33]. Unicellular cyanobacteria have round, oval, or cylindrical cells that can aggregate into irregular or regular colonies bound. The number of cells in each colony deviates from two to several thousand according to the species [33,37]. In the filamentous cyanobacteria forms, cells are connected to each other, forming a chain called “trichomes”. Branches and/or “pseudobranches” are formed when trichomes break or fragment [33].

Cyanobacteria are a boundless source of compounds and products of interest in the areas of health, food and feed, cosmetics, and energy production [38,39]. These include lipids, alkaloids, polyketides, peptides (lipopeptides, depsipeptides), vitamins, phenolic compounds, amino acids, polysaccharides, terpenes, and phycobiliproteins. Due to their rich bioactive content, cyanobacteria possess promising pro-health properties such as antioxidant, anti-inflammatory, antibacterial, antifungal, antidiabetic, anticancer, and neuroprotective activities [32,39].

## 3. The Beneficial Role of Cyanobacterial Natural Products in Inflammation and Oxidative Stress

Accruing evidence suggests the connection between inflammation, oxidative stress, and ND, like AD, PD, and ALS. Inflammation is the body’s natural response to infections, exposure to pathological health agents, chronic and autoimmune diseases, and unhealthy lifestyles [40]. This defense mechanism can take two stages: acute and chronic. Acute inflammation is the immune system’s first response, and it is usually temporary. When this defense mechanism fails, chronic and persistent inflammation occurs, leading to diseases such as diabetes, ND, and cancer [40]. Inflammation activates multiple cellular biochemical mediators, including cytokines such as interleukins (IL-1β and IL-6), tumor necrosis factor α (TNF-α), kinases, and transcription factors such as the nuclear factor κB (NF-κB) [41]. Nowadays, therapy for inflammation and inflammatory diseases is based on steroidal and non-steroidal anti-inflammatory drugs (NSAIDs). These NSAIDs act as prostaglandin inhibitors by inhibiting the cyclooxygenase (COX) enzymes. Despite their numerous benefits, long-term use of steroidal and non-steroidal drugs has been linked to hypertension, hyperglycemia, osteoporosis, and cardiovascular, gastrointestinal, and renal toxicity [41]. Therefore, the search for new therapies, namely those of natural origin, has become essential [42].

Neuroinflammation is the term used to describe inflammation of the CNS. This process occurs in response to endogenous or exogenous stimuli such as protein misfolding, mitochondria dysfunction, toxins, and pathogens [43]. During this defense mechanism, microglia and astrocytes are activated, leading to the production of inflammatory mediators such as cytokines and chemokines [44]. When these pro-inflammatory elements are overproduced, severe neurological damage can occur, resulting in neuronal death [45].

The use of neuroinflammation biomarkers is extremely important for disease prognosis. However, there are no specific biomarkers for various neurological diseases. Nevertheless, biomarkers for neuroinflammation include the translocator protein (18-kDa) (TSPO) and monoamine oxidase B (MAO-B) [43]. TSPO is a transmembrane protein located in the outer mitochondrial membrane [46]. In response to an inflammatory stimulus, the brain defense cells produce TPSO at higher levels. This biomarker is involved in several CNS functions, such as apoptosis regulation [47]. TSPO ligands have been demonstrated to be effective in reducing neuroinflammation and neuronal damage both in *in vivo* and *in vitro* models of neurodegenerative diseases. Neurosteroids’ production, cytokine release, and ROS metabolism are thought to be some of the events related to these findings [48].

Monoamine oxidase (MAO) enzymes regulate the amine levels in the brain [45]. These enzymes have two isoforms: monoamine oxidase A (MAO-A) and MAO-B, with the latter being predominant in brain glial cells [49]. MAO-B inhibitors, such as PD, are used to treat ND [49]. As astrocytes (glial cells) regulate MAO-B activity, it is thought that this enzyme can be used as a biomarker for neuroinflammation [45].

Reactive oxygen species are produced from molecular oxygen and are the result of cellular metabolism [50]. Superoxide anion (O_2_^•−^), hydrogen peroxide (H_2_O_2_), and hydroxyl radical (HO^•^) are some examples of this species [51]. ROS are mainly produced from the cytoplasmic membrane NADPH oxidase and from the mitochondrial respiratory chain [51].

The brain is very susceptible to oxidative stress since it consumes high amounts of molecular oxygen and has an elevated quantity of polyunsaturated fatty acids, which are very sensitive to peroxidation [51]. Most ND is associated with the atypical formation of protein aggregates, an event that can lead to oxidative stress due to mitochondrial dysfunction and ROS production [51]. Oxidative stress arises when there is an imbalance between the production of ROS and the capacity of antioxidant molecules to remove these radicals from the system. An antioxidant is a substance or molecule that can eliminate and/or prevent cell damage caused by ROS, which is produced in the system during physiological processes or can be related to external factors such as smoking, pollution, and radiation [52]. Excessive quantities of ROS can cause alterations in DNA molecules, lipids, and proteins. These modifications can lead to the development of diseases such as asthma, chronic inflammation, neurodegenerative and cardiovascular diseases, diabetes, and cancer [52].

In order to be protected against the harmful effects of ROS, human cells depend on their antioxidant capacity. Tissues incorporated antioxidant systems composed of various liposoluble (vitamin E, carotenoids) and hydrosoluble (ascorbic acid) components, and enzymatic machinery such as glutathione peroxidase (GSH), superoxide dismutase (SOD) and catalase (CAT) [29,53,54].

Most of the assays employed to assess the antioxidant potential of compounds or extracts rely on nonenzymatic assays. In those the most common ones include the 2,2′-azinobis-(3-ethylbenzothiazoline-6-sulphonate) radical (ABTS^•·+^) scavenging, the 1,1-diphenyl-2-picrylhydrazyl (DPPH^•^) radical scavenging, the Fe^3+^-Fe^2+^ transformation assay, the ferric reducing antioxidant power (FRAP) assay, the cupric ions (Cu^2+^) reducing power assay (Cuprac), O_2_^•·−^, H_2_O_2_ scavenging assay, ^•^OH scavenging assay, the singlet oxygen (^1^O_2_) quenching assay and ^•^NO· scavenging assay [55].

In the last decades, researchers have been investigating the anti-inflammatory and antioxidant properties of cyanobacterial natural products, focusing on extracts and pure compounds [32,50,51]. Most of the compounds described as potential anti-inflammatories were also found to induce antioxidant activity, and this includes polysaccharides, lipids, peptides, alkaloids, and pigments such as phycobiliproteins. A summary of these studies is presented in Table 1.

### 3.1. Polysaccharides

Polysaccharides are high molecular weight carbohydrates used by organisms as intracellular energy storage compounds, as structural cell wall components, or can be secreted as extracellular polymeric substances conferring protection against biotic and abiotic factors [93]. In cyanobacteria, most of the studied polysaccharides are extracellular polysaccharides that can be excreted into the surrounding medium, mostly referred to as exopolysaccharides (EPS), or remain more or less tightly bound to the cells forming capsules or mucilages. EPS from cyanobacteria are among the classes of compounds that have demonstrated anti-inflammatory and antioxidant effects in both in vivo and in vitro tests [94,95]. Cyanobacteria EPS are produced by several strains such as *Anabaena* spp., *Calothrix marchica*, *Cyanospira capsulate*, *Cyanothece* sp., *Leptolyngbya* sp., *Plectonema* sp., *Phormidium* sp. and *Nostoc* spp. These strains can produce, on average, up to >1 g/L of EPS, with *Nostoc* sp. CCALA H06/21 is the highest producer at around 8 g/L [96]. Zampieri et al. [56] extracted EPS from the cyanobacterium *Phormidium* sp. ETS05 and found that a concentration of 50 µg/mL EPS showed anti-inflammatory properties in chemical and physically injured zebrafish larvae by the reduction of NF-κB activity. Also, using the human skin fibroblast cell line HSF, the authors found that EPS concentrations ranging from 25 to 100 µg/mL increased cell viability.

Considering the antioxidant potential of polysaccharides, the antioxidant activity of polysaccharides extracted from the cyanobacterium *Nostoc commune* was assessed using several in vitro antioxidant assays (H_2_O_2_, O_2_^•−^, DPPH^•^, and FRAP). The DPPH^•^ assay showed the best results, with an EC_50_ of 2719.1 mg/L. Also, *Phormidium versicolor* polysaccharides were evaluated for their antioxidant activity in vitro, showing that the compounds strongly scavenged radicals, prevented bleaching of β-carotene, and reduced activity [57].

Capsular polysaccharides are natural polysaccharides strongly bound to the external cell wall surface [58]. Capsular polysaccharides and releasing polysaccharides from a methanolic extract of a *Leptolyngbya* sp. strain were examined for their antioxidant activities by means of DPPH, hydroxyl radical scavenging, and ferrous ion chelating assays [59]. Biomass methanolic extract presented both the highest DPPH and hydroxyl radical scavenging ability with an IC50 of 0.07 and 0. 38 mg/mL, respectively. The results of the ferrous ion chelating assays revealed that capsular polysaccharides methanolic extract presented the highest ferric chelating capacity with an IC 50 equal to 0.59 mg/mL. Also, capsular polysaccharides from *Nostoc flagelliforme* were found to improve the serum levels of inflammatory factors and antioxidant enzyme activity in the liver of male C57BL/6J mice [60]. From the same cyanobacteria species, Shen et al. [61] described the antioxidant potential of polysaccharides extracted from capsules. In this study, three polysaccharides (WL-CPS-1, NaCl-CPS-1, and Glu-CPS-1) were isolated. Antioxidant in vitro assays showed that WL-CPS-1, NaCl-CPS-1, and GluCPS-1 exhibited strong scavenging activity on ABTS+ and hydroxyl radicals. In fact, at the concentration of 2.5 mg/mL, the ABTS + radical scavenging abilities of WL-CPS-1, NaCl-CPS-1, and Glu-CPS-1 reached 89.54%, 91.62%, and 94.96%, respectively and the scavenging activities of hydroxyl radicals of WL-CPS-1, NaCl-CPS-1, and Glu-CPS-1 were 62.94%, 70.20% and 78.63%, respectively.

Sacran is a sulfated polysaccharide extracted from the cyanobacterium *Aphanothece sacrun* that has shown anti-inflammatory and antioxidant effects. Moytoma et al. [62] found that sacran inhibited paw edema induced by carrageenan, kaolin, and dextran, as well as ear edema induced by 12-O-Tetradecanoylphorbol-13-acetate (TPA) in male Wistar rats and female BALB/c mice. During the study, sacran inhibited edema in all the phlogistic agents at concentrations of 0.01% and 0.05% (*w*/*v*) for kaolin and 0.05% (*w*/*v*) for carrageenan, dextran, and TPA. Sacran cytotoxicity was studied in the HaCaT cell line, and no cytotoxic effects were observed. In another study, sacran reduced cell damage induced by sodium lauryl sulfate (SLS) and also restored the ROS levels stimulated by SLS and by Interleukin-1 alpha (IL-1α). The authors suggest that the anti-inflammatory and antioxidant effects of sacran are based on its trapping effect [63].

The antioxidant potential of sacran was also described by Doi et al. [64], in which sacran isolated from *Aphanotece sacrum* was tested in serum in human volunteers; a reduction in intracellular oxidation through decreases in IL-1α was observed, which indicated a reduction in oxidative stress.

### 3.2. Lipids

Cyanobacteria produce nonpolar and polar lipids that include glycerolipids, fatty acids, and their derivatives, as well as phospholipids and glycolipids, respectively. Also, cyanobacteria produce lipopeptides, which are amphiphilic molecules containing both a polar and an apolar moiety in their structure [97]. Glycolipids are a common type of lipid in cyanobacteria, serving as components of thylakoid membranes and heterocyst cell walls [98]. These molecules were discovered to induce anti-inflammatory activity [99]. Tena Pérez et al. [65] studied the effects of glycolipid fractions from *Nodularia harveyana* in lipopolysaccharide (LPS)- stimulated leukemic monocyte cells (THP-1) for their TNF-α and NF-κB inhibition. The fraction containing digalactosyldiacyl glycerols was the one with the highest inhibition for both factors, with an IC_50_ of 5.81 ± 0.23 μM for TNF-α and 3.75 ± 0.63 μM for NF-κB. This fraction also exhibited the lowest cytotoxicity (IC_50_ of 31.27 ± 0.12 μM).

In a study conducted by Al-Awadhi et al. [66], a monounsaturated fatty acid (7(*E*)-9-keto-hexadec-7-enoic acid) isolated from VPFK21-7 cyanobacterial mat presented anti-inflammatory and antioxidant activities. An ARE-luciferase reported assay was performed in HEK293 cells, and the compound was found to induce Nrf2 activity in a concentration of 10 and 32 µM, which demonstrates its antioxidant activity. The anti-inflammatory activity was tested by measuring the NO levels in LPS-activated mouse macrophage RAW 264.7 cells. The compound showed a decrease of NO levels in a dose-dependent manner due to a reduction in the inducible nitric oxide synthase (iNOs) transcript levels.

Lipidic factions of *Arthrospira subsalsa* exhibited strong anti-inflammatory properties in human platelets. These fractions strongly reduced platelet aggregation induced by platelet-activating factor (PAF), with IC_50_ values between 60 and 100 µg of polar lipids [67].

Moreover, *Gloeothece* sp. lipidic extracts were studied for their anti-inflammatory capacity by human red blood cell membrane stabilization and COX-2 screening assays and antitumor capacity by TUNEL assay in AGS cancer cells [68]. Several solvents (acetone, ethanol, ethyl lactate, and hexane: isopropanol) were tested to extract the lipidic compounds; the HI (3:2 hexane: isopropanol) appeared to be the most promising for use in the nutraceutical industry. HI extracts showed a 50% COX-2 inhibition with 130.2 ± 7.4 µg/mL; 61.6 ± 9.2% of lysosomes protection from heat damage and induced AGS cell proliferation up to 40% in a concentration of 23.2 ± 1.9 µg/mL. In the same study, the lipidic extract was also studied for its antioxidant activity by employing the ABTS^•+^, DPPH^•^, ^•^NO·, and O_2_^•−^ assays. HI extracts showed ^•^NO· radical scavenging capacity with an IC_50_ of 1258 ± 0.353 µg/mL [68].

Cyanobacteria produce a variety of chemically diverse lipopeptides, from linear lipopeptides to cyclic lipopeptides, that were found to induce anti-inflammatory and antioxidant properties [100]. The anti-inflammatory activity of the lipopeptides honaucins A–C isolated from a *Leptolyngbya crossbyana* strain was investigated by the NO production in LPS-stimulated macrophages RAW264.7 [69]. The NO production was inhibited by the compounds with IC_50_ values of 4.0, 4.5, and 7.8 μM, respectively. Honaucin A, in particular, was found to exert its anti-inflammatory activity through the activation of the cytoprotective nuclear erythroid 2-related factor 2 (Nrf2)-antioxidant response element/electrophile response element (ARE/EpRE) signaling pathway. The activation of this pathway was confirmed in cultured human MCF7 cells using an Nrf2 luciferase reporter assay [101].

**Figure 2:**
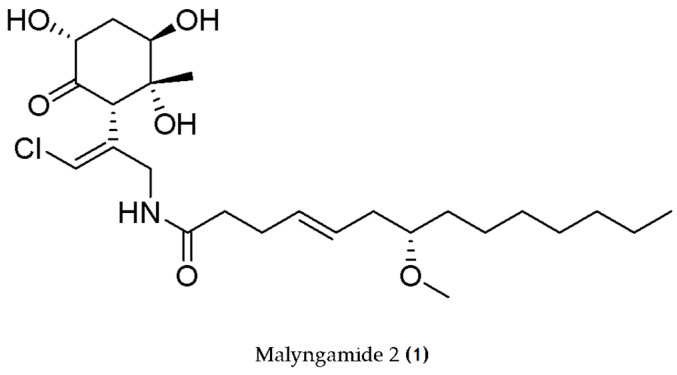
Structure of Malyngamide 2 (**1**), a lipopeptide that inhibits NO production.

In the same line, the oxidized lipopeptide malyngamide 2 (**1**) (Figure 2), isolated from *Lyngbya sordida*, displayed anti-inflammatory properties by inhibiting NO production with an IC_50_ = 8.0 μM in the murine RAW264.7 macrophage cell line treated with LPS [70].

### 3.3. Peptides

**Figure 3:**
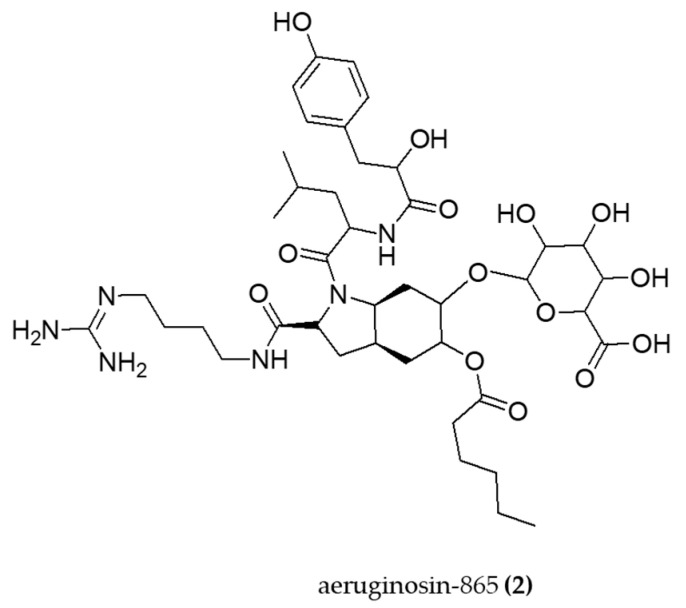
Structure of aeruginosin-865 (**2**) is a nonribosomal linear tetrapeptide that inhibits interleukin-8 (IL-8).

Peptides are the most abundant family of bioactive compounds produced by cyanobacteria [102]. Among these, the nonribosomal linear tetrapeptide aeruginosin-865 (**2**) (Figure 3) isolated from *Nostoc* sp. was shown to have pronounced anti-inflammatory effects. Kapuscik et al. [71] observed these findings using human lung microvascular endothelial cells (HLMVECs) stimulated with Human tumor necrosis factor-α (hTNF-α). Aeruginosin-865 inhibited interleukin-8 (IL-8) and intercellular adhesion molecule-1 (ICAM-1) levels in a dose-dependent manner, with EC_50_ values of 3.5 ± 1.5 µg/mL and 50.0 ± 13.4 µg/mL, respectively. The authors also discovered that this tetrapeptide partially or totally inhibits the translocation of the NF-κB dimer into the nucleus of endothelial cells.

Another two cyanobacterial peptides, Aeruginosin 828A and cyanopeptolin 1020, were found to have anti-inflammatory potential by decreased transcription of pro-inflammatory agents, like IL-8 and TNF-α in the TNF-α- simulated human liver cell line, Huh7 cells [72]. In addition, Ethyl tumonoate A isolated from *Oscillatoria margaritifera* revealed an anti-inflammatory effect through the NO assay without any cytotoxicity on mouse cell line RAW264.7 [73]. From *Arthrospira maxima,* two peptides, LDAVNR and MMLDF, isolated from the enzymatic hydrolysis of protein content, reduced the release of histamine and the production of IL-8 in response to histamine in endothelial cells [74].

### 3.4. Ultraviolet Absorbing Compounds

**Figure 4:**
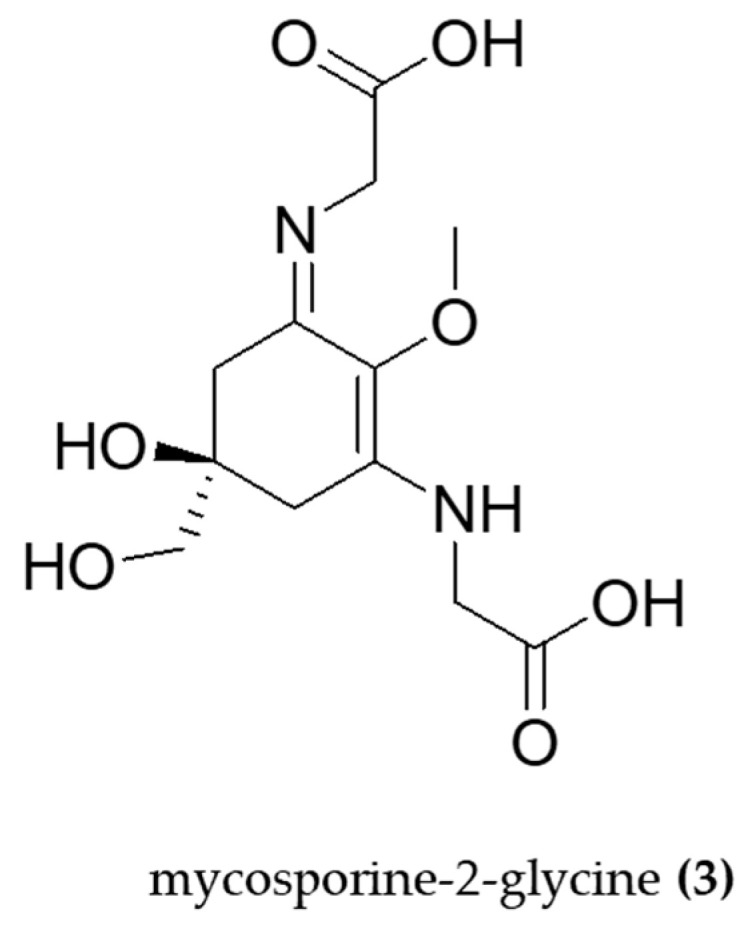
Structure of mycosporine-2-glycine (**3**) a Mycosporine-like amino acid able to inhibit iNOS and suppress the NF-κB pathway.

Mycosporine-like amino acids (MAAs) and scytonemin (SCY) are ultraviolet-absorbing compounds that help cyanobacteria to cope with UV radiation. Aside from this photoprotective effect, these compounds have been investigated as anti-inflammatories. The MAAs mycosporine-2-glycine (**3**) (Figure 4) extracted from *Aphanothece halophytica* was evaluated for its anti-inflammatory effects in LPS-induced RAW 264.7 macrophages. This compound was able to inhibit iNOS and suppress the NF-κB pathway at concentrations of 0.1–10 µM [75].

**Figure 5:**
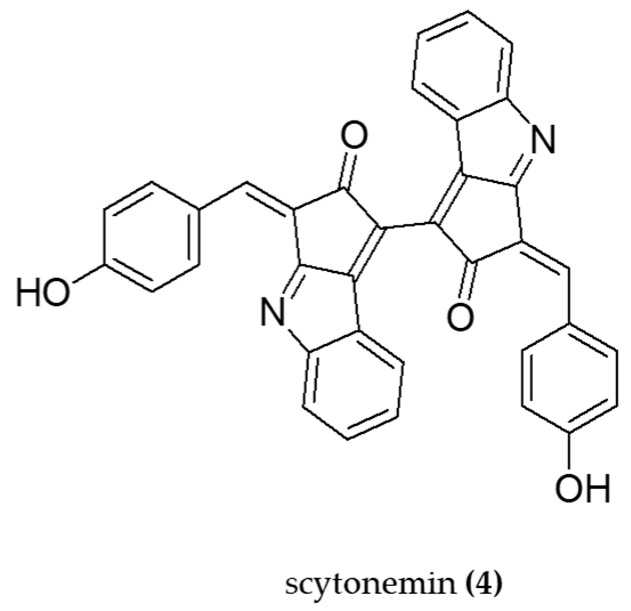
The structure of scytonemin (**4**) is an indole-alkaloid able to inhibit iNOS and suppress the NF-κB pathway.

Scytonemin (**4**) (Figure 5) is a yellow-green ultraviolet absorber pigment found in several genera of cyanobacteria [76]. This indole alkaloid demonstrated anti-inflammatory properties in both in vivo and in vitro studies [76]. In BALB/c mice, topical application of scytonemin (300 ng per ear) inhibited TPA-induced ear edema. It was also verified that TNF-α expression and iNos levels were suppressed in animal models. In addition, in LPS-stimulated RAW 264.7 macrophages, SCY (10–20 ng/mL) inhibited TNF-α and NF-κB expression.

Also for SCY, Rastogi et al. [77] reported significant inhibitory effects on ROS production in cyanobacterial cells treated with SCY (20.83%), ascorbic acid (AA) (45.75%), and SCY + AA (58.65%), after exposure to UVA 1 UVB 1, the photosynthetically active radiation. These results also indicate the potential role of SCY as a natural antioxidant.

### 3.5. Phycobiliproteins

Phycobiliproteins (PBP) are colored macromolecules that form part of the light-harvesting system in cyanobacteria. These water-soluble proteins can be classified according to their structure and light absorption properties as phycoerythrin, phycocyanin, phycoerythrocyanin, and allophycocyanin and are described as pigments with very interesting anti-inflammatory and antioxidant potential [103].

Phycobiliprotein extracts from *Cyanobium* sp. at a concentration of 100 µg/mL had the capacity to inhibit COX-1 and COX-2 in 50% and 40%, respectively. This extract also presented great antioxidant capacity [78].

C-phycocyanin (C-PC)isolated from *Arthrospira maxima* was tested for its anti-inflammatory and antiulcerogenic effects in rats with ethanol-induced gastric ulcers. Ethanol administration has been associated with ROS production. This was confirmed by the elevated malondialdehyde (MDA) concentration and decreased SOD, GSH, and CAT activity in rats treated with ethanol (96%, 5 mL/kg). In this group, TNF-α levels decreased in 54.10% and NF-κB levels in 28.37%. In contrast, in rats who received C-PC at 200 mg/kg, MDA activity was decreased, and SOD and CAT activities were increased [79]. Another in vivo study investigated the effects of oral C-PC (200 mg/kg) *Arthrospira platensis* on EAE (experimental autoimmune encephalomyelitis) induced Lewis rats. This administration reduced MDA, PP, and FRA levels while preventing myelin integrity. In the same study, treatment with phycocyanobilin (5 mg/kg) in EAE-C57BL/6 mice reduced neuroinflammation by lowering the expression of pro-inflammatory cytokines, IL-6, and IFN-γ [80].

C-phycocyanin, isolated from *Nostoc sphaeroides*, was studied in Doxorubicin (DOX) + C-PC induced C57BL/6 male mice in another study to investigate its neuroprotective activity. DOX is a drug commonly used in cancer treatment. However, it causes cognitive dysfunctions in patients. C-PC was shown to have neuroprotective effects mostly due to its antioxidant, anti-inflammatory, and mitochondrial properties. After several tests (MWM, quantification of TNF-α, IL-1β, IL-6, MDA, GSH, and SOD levels), the authors observed that C-PC treatment (50 mg/kg) suppressed all the neuronal problems caused by DOX treatment. In this sense, C-PC proves to be a good option choice for neuroinflammation and oxidative stress attenuation. As the registered anti-inflammatory potential, C-PC was shown to have neuroprotective effects, mostly due to its antioxidant properties. The authors observed that C-PC treatment (50 mg/kg) increases the activity of the antioxidant enzymatic system constituted by GSH and SOD [81].

Phycocyanin extracted from the cyanobacterium *Geitlerinema* sp. was investigated for its antioxidant activity [82]. Several methods were performed, such as phosphomolybdenum assay, DPPH^•^, H_2_O_2_, FRAP, and anti-lipid peroxidation assay. At 200 µg/mL, the blue pigment had a maximum absorbance of 0.49 nm by phosphomolybdenum assay, 0.85 nm absorbance by FRAP assay, 78.75% DPPH^•^ scavenging activity, 95.27% H_2_O_2_ scavenging activity and in the anti-lipid peroxidation assay an activity of 53.65%. These findings suggest that C-PC acts as a promising antioxidant compound with potential for use in oxidative stress-related diseases.

Phycoerythrin (PE) extract from *Halomicronema* sp. R31DM demonstrated excellent in vivo and in vitro antioxidant activity [83]. A decrease in ROS levels was registered in a *Caenorhabditis elegans* model. In this in vivo test, *C. elegans* worms were exposed to an atmosphere of high temperature and strong oxidizing agents. Worms fed with PE (100 µg/mL) showed a higher survival rate (72%) compared to the control group. Also, the worms were exposed to 10 mM paraquat solution, which induces ROS production. ROS levels were measured with DCFH-DA staining. Fluorescence was measured, and the groups fed with PE showed no fluorescence, indicating PE’s ROS scavenging potential. The in vitro antioxidant activity of PE was measured by the DPPH^•^, FRAP, and RP (reducing power) assays. In the DPPH^•^ assay, PE showed a scavenging activity of 64% at 100 µg/mL. Ascorbic acid, the positive control used in this assay, showed a 100% scavenging activity at the same concentration. The FRAP assay demonstrated a direct relation between the absorbance and the increasing PE concentration. Regarding the RP assay, it showed a dose-dependent increase in the OD at 700 nm.

### 3.6. Carotenoids and Phenolic Compounds

Carotenoids are tetraterpenoid molecules present in all photosynthetic organisms, namely cyanobacteria, from which rich carotenoid extracts exert anti-inflammatory and antioxidant properties [104]. Phenolic compounds are also common in cyanobacteria and are also responsible for anti-inflammatory and antioxidant properties [105].

Several works on the anti-inflammatory and antioxidant potential of cyanobacteria were performed in characterized extracts for carotenoid and phenolic content [106]. *Lyngbya* sp. and *Oscillatoria* sp. extracts were characterized for their total phenolic content (TPC), total flavonoid content (TFC), and PBP. *Lyngbya* sp., showed TPC values of 5.02 ± 0.20 mg/g, TFC of 664.07 ± 19.76 mg/g, and total PBPs of 127.01 mg/g. When these two species were compared in terms of antioxidant potential, the FRAP assay was higher in *Oscillatoria* sp. (39.63 ± 7.02 µM Fe [II]/100 g). DPPH radical scavenging activity was also highest in *Oscillatoria* sp. (465.31 ± 25.76 mg/g). The authors suggest that these two cyanobacteria strains may be good sources of antioxidants with special use in the food and pharmaceutical industry [84].

The freshwater *Aphanothece microscopica Nägeli* (RSMan92) [85], the marine *Cyanobium* sp. LEGE 06113 [78] and the marine *Trichodesmium* sp. [86] are also excellent sources of carotenoids. In *Aphanothece microscopica,* the highly potent antioxidant all-trans-b-carotene and small amounts of 9-cis-b-carotene and retinyl palmitate were detected and described as responsible for the antioxidant potential of the strain in non-polar and polar organic extracts with mean FRAP values of 66.9 ± 0.25 and 41.7 ± 1.2 mM per mg crude organic extract in the non-polar and polar organic extracts. In *Cyanobium* sp. LEGE 06113, the bioactive potential was assessed for antioxidant capacity by ABTS^•+^, ^•^NO, and O_2_^•−^ scavenging assays and anti-inflammatory capacity by COX inhibition assay. In this cyanobacteria strain, W-A showed a higher antioxidant capacity and higher content of carotenoids. In terms of anti-inflammatory capacity, 100 μg_E_ mL^−1^ of E-W extract exhibited the capacity to inhibit both COX-1 and COX-2 enzymes *Trichodesmium* sp.

## 4. *Arthrospira* Studies of Success

*Arthospira* species are attractive producers of many valuable compounds useful for food, feed, and pharmaceutical industries, namely due to their anti-inflammatory and antioxidant effects [107,108]. Several research works point to *Arthospira* as a potential case of success in neuroprotection, as described below.

In a study by Chen et al., [28] it was found that *Arthrospira* and C-PC reduced cytotoxicity and inflammation-related gene expression of microglial cells. In BV-2 microglial cells treated with LPS (1 μg/mL) and *Arthrospira platensis* water extract and C-PC, a significant reduction in LDH release occurred along with the reduction in the expression of iNOS, COX-2, TNF-α, and IL-6 mRNAs. Thus, it was hypothesized that *A. platensis* inhibits the expression of inflammation-related genes of LPS-stimulated BV-2 microglial cells. These findings can open the hypothesis that orally administered *A. platensis* or C-PC may provide protection from NDs in which microglia cells play a pathogenic role.

Also working with microglia activation in neurodegeneration, Piovan, and co-authors studied the effect of an *A. platensis* acetone extract on the release of IL-1β and TNF-α, expression of iNOS, nuclear factor erythroid 2–related factor 2 (Nrf2) and the activation of NF-κB in primary microglia firstly stimulated with LPS [87]. Results indicated that the extract downregulated the release of IL-1β and TNF-α and led to the over-expression of iNOS. The extract blocked the LPS-induced nuclear translocation of NF-κB p65 subunit and upregulated gene and protein levels of Nrf2, as well as gene expression of HO-1, indicating a control in microglia activation and thus protection against neuroinflammation.

A non-protein extract from *A. platensis* was also tested for neuroprotective properties using PC12 (pheochromocytoma) cells [88]. Abnormal iron accumulation in the brain has been pointed out as one of the causes of oxidative damage and neuronal cell death. The ferric-reducing antioxidant potency and the free radical scavenging activity were assessed. These assays showed good results at concentrations between 5 and 50 mg/mL. The authors conclude that *A. platensis* has properties that can be effective against oxidative damage in neuronal cells.

In an in vivo study using DJ-1β^∆93^ flies, a PD model in *Drosophila*, exposed to paraquat to induce oxidative stress, *Arthrospira* supplementation (5% or 10%) and C-PC (1 or 2 µg/mL) reduced cellular stress and showed antioxidant effects. A diet with *Arthrospira* increased the lifespan and locomotor activity in the flies and downregulated SOD and CAT activity [89].

Koh et al. [90] used a 70% *Arthrospira maxima* ethanol extract (SM70EE, 100 µg/mL) in order to investigate its effects against Aβ_1–42_- induced neurotoxicity in PC12 cells. High levels of Aβ induce oxidative stress in the brain and neuronal cell death. Also, Aβ stimulates the cleavage of poly (ADP-ribose) polymerase (PARP). Overactivation of PARP is related to cell death and neuroinflammation. Neurotoxicity was induced with 4 µg/mL of Aβ_1–42_. The results showed that SM70EE prevented PC12 cell death and reduced PARP cleavage. Oxidative stress was reduced, and GSH levels were restored.

In another study, SM70EE was used in trimethyltin (TMT, 10µM)- induced HT-22 cells and scopolamine (1 mg/kg body weight/day)-induced ICR (Institute of Cancer Research) mice [91]. TMT induces neuronal cell apoptosis, is related to oxidative stress and mitochondrial and neurotransmitter dysfunction, and increases AChE activity. Once again, PARP cleavage was inhibited, and ROS production was decreased in the presence of the extract (50 and 100 µg/mL). Moreover, AChE activity was blocked with SM70EE. Regarding the in vivo tests, oral administration of 200 and 400 mg/kg body weight/day of SM70EE prevented learning and memory damage related to scopolamine-induced neurotoxicity.

A 70% ethanolic extract of *A. maxima* was tested in mouse RAW264.7 macrophages, previously treated with LPS [109]. The results indicated that the extract suppressed LPS-induced upregulation of the pro-inflammatory cytokines tumor necrosis factor-α, interleukin (IL)-12, IL-1β, and IL-18 in RAW264.7 and attenuated the generation of ERK1-induced ROS, resulting in decreased expression of NF-κB, highlighting its anti-inflammatory and antioxidant potential.

In a study by Angelica et al., [30] the neuroprotective effect of *A. platensis* against kainic acid neuronal death was evaluated. In this study, male SW mice were treated with *A. platensis* for 24 days, at doses of 0, 200, and 800 mg/kg, once daily, and with kainic acid (35 mg/kg, ip) as a single dose on day 14. The authors found that a pretreatment reduces mice mortality and neurobehaviour improvement, which was correlated with the capacity of *A. platensis* to reduce kainic acid -neuronal death in CA3 hippocampal cells. The authors believe that the neuroprotection may be related to the antioxidant properties of *A. platensis*.

In a comparative study of the anti-inflammatory and antioxidant activity of methanolic extracts from *A. platensis* and *A. maxima* strains in RAW 264.7 macrophages stimulated with LPS, it was described a reduction in NO release and a suppression in iNOS and COX-2 expression by *A. platensis,* while *A. maxima* lacked effects [110]. However, *A. maxima* showed the highest inhibition of NLRP3 and IL1-β expression and IL1-β secretion. It also found that both strains displayed direct antioxidant activity and counteracted LPS-induced SOD2 overexpression without affecting HO-1 expression.

Still considering *Arthrospira*, tablets of *A. platensis* (1500 mg/kg) showed neuroprotective potential in aluminum chloride (AlCl_3_) induced Wistar rats. The treatment reduced TNF-α, indicating anti-inflammatory activity. It also showed high antioxidant potential by restoring GSH levels, thiol content, and total antioxidant capacity (TAC) [92].

## 5. Opportunities, Prospects, and Research Directions

The quest for novel anti-inflammatory and antioxidant compounds grips special attention in neurodegeneration research and development. Anti-inflammatories are pivotal in reducing inflammation, and antioxidants are crucial in neutralizing oxidative stress, which is linked to the neurodegenerative process.

The increasing interest in natural compounds to fight inflammation and oxidative stress is highly linked with the potential toxicity associated with synthetic ones to both human health and the environment [111]. The awareness in investigating these activities in cyanobacteria stems from their rich list of compounds, including polysaccharides, lipids, pigments, carotenoids, and polyphenols, to the sustainability in the production of biomass and extraction procedures. In fact, the growing awareness of the health benefits of natural compounds allied with the World Health Organization (WHO) Intersectoral global action plan on epilepsy and other neurological disorders emphasizing diets enriched with natural compounds is an opportunity that drives more research in the biotechnological potential of cyanobacteria [79,80]. Moreover, the forthcoming climate crisis has encouraged an interest in cyanobacteria not only due to their potential source of natural compounds but also to the sustainability of biomass production and extraction [112].

As photosynthetic organisms, cyanobacteria energy production relies on water, CO_2,_ and sunlight, making these organisms a promising alternative for carbon sequestration, which provides an efficient and feasible strategy to sequester excess carbon. Cyanobacteria show a higher photosynthetic efficiency of up to 10% than terrestrial plants and, in addition, require minimal land and nutrient input [113]. Also, the major advantages of cyanobacteria in neuroprotection come from the fact that the same strain can produce both anti-inflammatory and antioxidant compounds, improve disease onset, and improve behavior and lifespan, as reviewed by Ramos et al. [114].

A successful example of the use of cyanobacteria for related health purposes is the genus *Arthrospira* and *Nostoc*. *Arthrospira* is a well-known and largely consumed cyanobacterium, and *Nostoc* is widely used as food in Asia and South America [83,104]. At present, more than 70 countries have commercialized products of nutritional importance that are obtained from cyanobacteria [115]. *Athrospira maxima* and *Arthrospira platensis* are edible cyanobacteria commonly cultivated for their protein content, carbohydrates and vitamins (A, C and E), minerals (iron, calcium, chromium, copper, magnesium, manganese, phosphorus, potassium, sodium and zinc), essential fatty acids, carotenoids (β-carotene), chlorophyll-a, and PBS (C-PC and allophycocyanin) and globally commercialized as dietary supplements in forms as powder, capsules and tablets [83,84,106]. *Nostoc sphaeroides* is an edible cyanobacterium with high nutritional value and is widely used in dietary supplements and therapeutic products. As described, *N. sphaeroides* contains protein, minerals, vitamins, polysaccharides, PBP, and some lipids with high bioactive potential, namely in antioxidation and anti-inflammation-reducing functions [116]. For example, the PBP, namely C-PC isolated from *N. sphaeroides*, proved to be a good option for neuroinflammation and oxidative stress attenuation [57].

Notwithstanding the industrial interest in cyanobacteria, many species are still underexploited, mainly due to lower production yields. However, the development of industrial culture systems, namely large-scale production of biomass and the improvement of downstream processes, associated with increased knowledge about factors affecting production, could support the development of the market chain. Additionally, with the current availability of the genome sequences and metabolic models of several cyanobacteria strains, the development of cell factories can now be accelerated by means of genetic and metabolic engineering approaches directing cyanobacteria to produce specific compounds.

## 6. Conclusions

Cyanobacteria proved to be a promising source of anti-inflammatory and antioxidant compounds. Their minimal growth requirements, higher efficiency of photosynthesis and growth rates, presence of considerable amounts of different bioactive compounds, cosmopolitan nature, and the ability to culture on non-arable land make these organisms a safe and sustainable alternative to producing compounds with a potential focus on neurodegeneration. Cyanobacteria are a promising candidate to act as a new approach to the treatment of ND. Nevertheless, their use has yet to be fully explored, and further research is needed, especially in human clinical trials. However, the commitment to exploring safer and sustainable alternatives such as cyanobacteria presents an opportunity for curing or preventing this type of disease.

## Figures and Tables

**Table 1:** Cyanobacteria in inflammation and oxidative stress.

Genus/Species/Code	Compound/Extract	Mechanism/Effect	In Vitro Assays	In Vivo Assays	Reference
*Phormidium* sp. ETS05	Exopolysaccharides (EPS)	Anti-inflammatory	Human skin fibroblasts (HSF) exposed to EPS	Chemical (copper and DSS) and injury (amputation) zebrafish larvae	[56]
*Nostoc commune*	Polysaccharides	Antioxidant Antibacterial	H_2_O_2_, O_2_^•−^, DPPH^•^, RP	-	[57]
*Phormidium versicolor*	Polysaccharides	Antioxidant Antimicrobial	DPPH, FRAP, β-carotene bleaching, HO^•^ assays	-	[58]
*Leptolyngbya* sp.	Polysaccharides	Antioxidant	DPPH, HO^•^, Fe^2+^	-	[59]
*Nostoc flagelliforme*	Capsular polysaccharides	Antioxidant Anti-inflammatory	-	Male C57BL/6J mice	[60]
*Nostoc flagelliforme*	Polysaccharides	Antioxidant	DPPH, ABTS^+^, HO^•^ assays	-	[61]
*Aphanothece sacrum*	Sacran	Anti-inflammatory.	HaCaT human keratinocyte cells. WST-1 method	Male Wistar rats and female BALB/c mice with paw edema induced by carrageenan, kaolin and dextran and ear edema induced by TPA. Histological analysis.	[62]
*Aphanothece sacrum*	Sacran	Anti-inflammatory. Antioxidant	HaCaT keratinocytes cells. ROS induced by SLS and IL-1α.	-	[63]
*Aphanothece sacrun*	Polysaccharide	Anti-inflammatory	-	Topical treatment with sacrun-containing serum in human volunteer (size, percentage of thick abrasion, ratio of SH to SS groups, ratio of IL-1 receptor antagonist to IL-1a, and carbonylated protein level).	[64]
*Nodularia harveyana*	Glycolipids	Anti-inflammatory Downregulation of TNF-α and NF-κB	LPS-stimulated leukemic monocyte cells (THP-1)	-	[65]
VPFK21-7	Monounsaturated fatty acid 7(E)-9-keto-hexadec-7-enoic acid	Anti-inflammatory	HEK293 ARE-luc cells (ARE-Luciferase Reporter Assay) RAW264.7 cells (^•^NO levels)	-	[66]
*Arthrospira subsalsa*	Lipid fractions	Anti-inflammatory	hPRP (anti-PAF, antohrombotic activities)	-	[67]
*Gloethece* sp.	Lipidic extracts	Antioxidant Anti-inflammatory Anti-tumor	ABTS^•+^, DPPH^•^, ^•^NO, O_2_^•−^ HRBC, COX-2 TUNEL in AGS cancer cells	-	[68]
*Leptolyngbya crossbyana*	Lipopetides (Honaucins A-C)	Anti-inflammatory	LPS-stimulated RAW264.7 cell line (^•^NO production)	-	[69]
*Lyngbya sordida*	Lipopeptide (Malyngamide)	Anti-inflammatory	LPS-induced RAW 264.7 cell line (^•^NO production)	-	[70]
*Nostoc*	Aeruginosin-865	Anti-inflammatory NF-κB inhibition	hTNF-α–stimulated HLMVECs cells (AlphaLISA assay). IL-8 and ICAM-1 levels	-	[71]
*Planktothrix rubescens*	Aeruginosin 828A and cyanopeptolin 1020	Anti-inflammatory	Human hepatoma cell line Huh7 (IL-8, TNF-α)	Zebrafish AG 828A expoused	[72]
*Oscillatoria margaritifera*	Ethyl tumonoate A	Anti-inflammatory	Mouse cell line RAW264.7 (^•^NO assay) Neocortical neurons from Swiss webster mice (intracellular Ca^2+^ monitoritation)	-	[73]
*Arthrospira maxima*	Peptides (LDAVNR and MMLDF)	Anti-inflammatory	Antigen-stimulated RBL-2H3 mast cells. Histamine-stimulated EA.hy926 endhothelial cells	-	[74]
*Aphanothece halophytica*	Mycosporine-2-glycine	Anti-inflammatory Antioxidant	LPS- induced macrophages (RAW 264.7). iNOS, NF-κB, COX-2, H_2_O_2_.	-	[75]
*-*	Scytonemin	Anti-inflammatory	LPS-stimulated RAW 264.7 cells (TNF-α and NF-κB)	BALC/c mice with TPA-induced ear edema (TNF-α, iNOS)	[76]
*Scytonema* sp. R77DM	Scytonemin	Antioxidant	Cyanobacterial cells treated with scytonemin (ROS production)	-	[77]
*Cyanobium* sp.	Phycobiliproteins and carotenoids	Anti-inflammatory Antioxidant	ABTS^•+^, ^•^NO, O_2_^•−^ scavenging COX inhibition		[78]
*Arthrospira maxima*	C-phycocyanin	Anti-inflammatory Anti-ulcerogenic	-	Male Wistar rats with ethanol-induced gastric ulcers (MDA, GSH, SOD, CAT, TNF-α, NF-κB)	[79]
*Arthrospira platensis*	C-Phycocyanin	Antioxidant Anti-inflammatory Neuronal protection	-	EAE indued male Lewis rats and female C57BL/6 mice: MDA assay. PP assay. FRA assay. ELISA (IL-17, IL-6, IFN-γ).	[80]
*Nostoc sphaeroides*	C-phycocyanin	Anti-inflammatory Antioxidant Mitochondria protection Synapse protection	-	DOX + CP- induced C57BL/6 male mice (MWM, TNF-α, IL-1β, IL-6, MDA, GSH and SOD levels)	[81]
*Geitlerinema* sp. TRV57	Phycocyanin	Antioxidant	Phosphomolybdenum, DPPH^•^, H_2_O_2_, FRAP, Antilipid peroxidation assays	-	[82]
*Halomicronema* sp. R31DM	Phycoerythrin	Antioxidant	DPPH, FRAP, RP	N2 Bristol wild type *C. elegans* (ROS levels)	[83]
*Lyngbya* sp. and *Oscillatoria* sp.	Phycobiliproteins, Phenolic and Flavonoids compounds	Antioxidant	TPC, TFC, PBPs, FRAP and DPPH^•^	-	[84]
*Aphanothece microscopica Nageli*	Carotenoids	Antioxidant	ROO^•^ scavenger capacity	-	[85]
*Trichodesmium* sp.	Carotenoids	Anti-inflammatory	COX-1 and COX-2 inhibition	-	[86]
*Arthrospira platensis*	Acetonic extract	Anti-inflammatory	Primary microglia (IL-1β, TNF-α, iNOS, Nrf2, HO-1)		[87]
*Arthrospira platensis*	Non-protein extract	Antioxidant	PC 12 cells (Ferric- reducing antioxidant activity and DPPH^•^)	-	[88]
*Arthrospira platensis*	Diet supplementation (5 and 10% *w*/*v*)	Antioxidant Reduced cellular stress	-	DJ-1β∆93 Drosophila Melanogaster exposed to paraquat: Survival assay. Locomotor assay. Enzymatic assays (SOD and CAT).	[89]
*Arthrospira maxima*	70% ethanol extract (SM70EE)	Neuroprotection Antioxidant	Aβ_1–42_- induced PC12 cells (MTT, PARP, LDH, GSH levels, western blot)	-	[90]
*Arthrospira maxima*	70% ethanol extract (SM70EE)	Antioxidant AChE inhibition	TMT- induced HT-22 cells (MTT, PARP, western blotting)	Scopolamine-induced ICR mice (MWM, Passive avoidance test)	[91]
*Arthrospira platensis*	Diet supplementation (1500 mg/kg, tablets)	Antioxidant Anti-inflammatory Neuronal morphology protection	-	Wistar rats induced with AlCl3: GSH content assay. Total thiol content assay. TAC assay. ELISA (TNF-α). Histology. Immunofluorescence (Aβ).	[92]

Abbreviations: NF-κB—Nuclear factor κB; hTNF-α—human tumor necrosis factor α; HLMVECs—human lung microvascular endothelial cells; IL-8—Interleukin-8; ICAM-1—intercellular adhesion molecule 1; WST-1—water-soluble tetrazolium salt; TPA—12-*O*-Tetradecanoylphorbol13-acetate; HEK293 ARE-luc—human embryonic kidney cells stably transfected with firefly luciferase reporter gene; ARE—antioxidant response element; NO—nitric oxide; ABTS—2,2′-Azino-bis (3-ethylbenzothiazoline-6-sulfonic acid; DPPH—2,2-diphenyl-1-picrylhydrazyl; ^•^NO—nitric oxide; O_2_^•−^—Superoxide anion; HRBC—Human red blood cell; COX-2—cyclooxygenase-2; TUNEL—Terminal deoxynucleotidyl transferase dUTP nick-end labeling; AGS—gastric adenocarcinoma cell-line; H_2_O_2_—Hydrogen peroxide; RP—reducing power; DOX—Doxorubicin; CP—C-phycocyanin; MWM—Morris water maze; TNF-α—Tumor necrosis factor alpha; IL-1β—Interleukin 1 beta; IL-6—Interleukin-6; MDA—Malondialdehyde; GSH—Glutathione; SOD—Superoxide dismutase; FRAP—Ferric ion reducing ability of plasma; *C. elegans*—*Caenorhabditis elegans*; PC 12—Pheochromocytoma; MTT—3-(4,5-dimethylthiazol-2-yl)-2,5-diphenyl tetrazolium bromide; PARP—poly (ADP-ribose) polymerase; LDH—lactate dehydrogenase; ICR—institute of cancer research; CAT—catalase; hPRP—human platelet-rich plasma; PAF—platelet-activating factor; SLS—sodium lauryl sulfate; HSF—human skin fibroblasts; DSS—Dextran sulfate sodium; TMT—trimethyltin; SH—sulfhydryl; SS—disulfide; ROO^•^—Peroxyl radical; Nrf2—nuclear factor erythroid2-related factor; HO-1—heme oxygenase-1.

## Data Availability

No new data were created or analyzed in this study.
